# Q Fever in Pregnant Goats: Pathogenesis and Excretion of *Coxiella burnetii*

**DOI:** 10.1371/journal.pone.0048949

**Published:** 2012-11-09

**Authors:** Hendrik-Jan Roest, Betty van Gelderen, Annemieke Dinkla, Dimitrios Frangoulidis, Fred van Zijderveld, Johanna Rebel, Lucien van Keulen

**Affiliations:** 1 Department of Bacteriology and TSEs, Central Veterinary Institute, part of Wageningen University and Research Centre, Lelystad, The Netherlands; 2 Bundeswehr Institute of Microbiology, Munich, Germany; 3 Department of Infection Biology, Central Veterinary Institute, part of Wageningen University and Research Centre, Lelystad, The Netherlands; East Carolina University School of Medicine, United States of America

## Abstract

*Coxiella burnetii* is an intracellular bacterial pathogen that causes Q fever. Infected pregnant goats are a major source of human infection. However, the tissue dissemination and excretion pathway of the pathogen in goats are still poorly understood. To better understand Q fever pathogenesis, we inoculated groups of pregnant goats via the intranasal route with a recent Dutch outbreak *C. burnetii* isolate. Tissue dissemination and excretion of the pathogen were followed for up to 95 days after parturition. Goats were successfully infected via the intranasal route. PCR and immunohistochemistry showed strong tropism of *C. burnetii* towards the placenta at two to four weeks after inoculation. Bacterial replication seemed to occur predominantly in the trophoblasts of the placenta and not in other organs of goats and kids. The amount of *C. burnetii* DNA in the organs of goats and kids increased towards parturition. After parturition it decreased to undetectable levels: after 81 days post-parturition in goats and after 28 days post-parturition in kids. Infected goats gave birth to live or dead kids. High numbers of *C. burnetii* were excreted during abortion, but also during parturition of liveborn kids. *C. burnetii* was not detected in faeces or vaginal mucus before parturition. Our results are the first to demonstrate that pregnant goats can be infected via the intranasal route. *C. burnetii* has a strong tropism for the trophoblasts of the placenta and is not excreted before parturition; pathogen excretion occurs during birth of dead as well as healthy animals. Besides abortions, normal deliveries in *C. burnetii*-infected goats should be considered as a major zoonotic risk for Q fever in humans.

## Introduction

*Coxiella burnetii* is a Gram-negative intracellular bacterium and the causative agent of Q fever. *C. burnetii* can affect a wide range of hosts, including humans, ruminants, companion animals, birds and reptiles [1]. *C. burnetii* is present throughout the world, with the exception of New Zealand [2]. The bacterium is considered a biothreat agent in view of its very low infectious dose and high transmissibility [3]. Clinical infection in humans manifests as atypical pneumonia, hepatitis or flu-like self-limiting disease. Persistent infection may result in life-threatening endocarditis. The main infection route is via inhalation of *C. burnetii*-contaminated aerosols from the environment [4]. Excretion of *C. burnetii* by domestic ruminants is considered the source of environmental contamination and the cause of human infection [5], [6], [7]. The zoonotic impact of Q fever was recently underlined by the Dutch Q fever outbreak involving >4000 registered human cases in regions with high frequencies of Q fever abortions in dairy goats [8], [9]. Molecular typing of *C. burnetii* isolates confirmed the epidemiological link between human and animal infections [10], [11], [12].

A key factor in the zoonotic transmission of the Q fever agent is the excretion of the pathogen by the animal host. Animals are assumed to become infected by inhalation or oral uptake of *C. burnetii* from the environment. *C. burnetii* infection in animals is generally asymptomatic. In pregnant animals, however, *C. burnetii* infection can become symptomatic. Metritis, abortion, stillbirth and delivery of weak offspring are the most frequent clinical signs of disease [13]. In symptomatic individuals *C. burnetii* is excreted via faeces, vaginal mucus, milk and birth products. In pregnant goats the clinical manifestation of *C. burnetii* infection is abortion and stillbirth in the final stage of gestation. Although transmission from goats to humans is assumed to occur after abortion resulting from Q fever infection, field studies suggest that *C. burnetii* may also be excreted via the placenta during normal parturition [14], [15], [16], [17].

The excretion of *C. burnetii* in infected pregnant goats via faeces, vaginal mucus and milk is poorly understood. Excretion via these routes was confirmed in an experimental setup [18], but field observations revealed a poor correlation between the routes [17]. Excretion via faeces, vaginal mucus and milk suggest that the pathogen is disseminated towards different bodily fluids during infection. Evidence for this is limited. After subcutaneous inoculation of pregnant goats, *C. burnetii* DNA was shown to be present in the mammary glands, uterus, liver, spleen and lungs at one or both of the two investigated points in time between inoculation and abortion [19]. Whether the presence of *C. burnetii* resulted in its excretion was not investigated. The influence of the infection route on dissemination and excretion of the pathogen has not been investigated in goats either.

The goal of the present study was to systematically monitor the dissemination and excretion of *C. burnetii* in pregnant goats before and after parturition following inoculation of the agent via a natural inoculation route. Bacterial dissemination and excretion and the development of pathology were followed using a combination of microbial and DNA detection techniques, post-mortem examination and immunohistochemistry. Our results indicate that the placenta is the primary target organ and infection source of *C. burnetii* in pregnant goats. *C. burnetii* is not excreted before parturition and excretion can occur both during abortion as well as during delivery of healthy newborns.

## Results

### Comparison of *Coxiella burnetii* Infection Routes in Goats

To investigate natural routes of *C. burnetii* infection in goats, we compared the effectiveness of intranasal and oral routes with the subcutaneous inoculation route in pregnant Dutch dairy goats. Two goats per dose-route combination were given either an oral or intranasal dose of 10^4^ or 10^6^ MID, or a subcutaneous dose of 10^4^ MID. The infection outcome was monitored by the occurrence of abortion and testing the placenta for the presence of *C. burnetii* DNA using PCR.

At the start of the experiment, several of the goats were in an obese condition. The goats had a poor appetite, probably due to adaptation problems from the herd diet to the diet fed in the experimental facilities. We observed three early abortions, one in the subcutaneously inoculated group (abortion at 12 dpi; placenta *C. burnetii* negative), one in the 10^6^ orally challenged group (abortion at 13 dpi; placenta *C. burnetii* negative) and one in the 10^4^ orally challenged group (abortion at 21 dpi; placenta *C. burnetii* positive). Two goats had to be euthanised before the end of the experiment for ethical reasons: one in the control group (at 41 dpi) and one in the 10^4^ orally challenged group (at 62 dpi). In the intranasally inoculated group three abortions were observed and the placentas were strongly positive for *C. burnetii* DNA. In the orally inoculated group one goat gave birth to two kids and no *C. burnetii* DNA could be detected in the placenta, and one goat was euthanised at the end of pregnancy and low amounts of *C. burnetii* DNA were detected in the placenta (Ct value between 30 and 40). The results are detailed in Table 1. Overall, inoculation via the nasal route was most successful. It resulted in abortion at earlier time points after inoculation and a more heavily infected placenta as compared to infection via the oral and subcutaneous routes.

**Table 1:** Results of the comparison of the subcutaneous, oral and nasal infection route in pregnant goats with two doses of *Coxiella burnetii*.

Inoculation route	dose (MID)	goat	pregnancy outcome	dpi	PCR results placenta
None	none	1	euthanasia	41	−
		2	kidding	63	−
Oral	10^Λ^4	1	abortion	21	+
		2	euthanasia	62	+
Oral	10^Λ^6	1	abortion	13	−
		2	kidding	64	−
Nasal	10^Λ^4	1	abortion	48	+++
		2	mummification	88	+
Nasal	10^Λ^6	1	abortion	53	+++
		2	abortion	54	+++
Subcutaneous	10^Λ^4	1	abortion	12	−
		2	1 dead/1 alive	61	+++

MID: mouse infective dose; dpi: days post inoculum, “−”: negative result, “+”: 30≤PCR cycle threshold (Ct)<40, “++”: 20≤Ct<30, “+++”: Ct<20.

### Intranasal Infection and Pregnancy Outcome

In subsequent challenge experiments (II and III) we used first-parity pregnant goats raised in experimental facilities rather than Dutch goats raised in a commercial dairy farm to limit the problems associated with the adaptation of the goats to the experimental facilities. Goats were inoculated on day 76 of pregnancy via the intranasal route with 10^6^ MID of the Dutch outbreak *C. burnetii* strain. Control goats were intranasally inoculated on day 76 of pregnancy with culture medium. In both experiments animals did not show any clinical signs of disease except for the occurrence of abortions in the Coxiella-inoculated groups. The goats’ appetite was good from the start and no deviation from the normal rectal temperature of goats (38.5–40.0°C) was measured for more than one day in either the control group or the Coxiella-inoculated group.

In Experiment II, seven Coxiella-inoculated goats gave birth. *C. burnetii* was detected by PCR and IHC in all placentas, indicating successful intranasal inoculation in all animals. Three goats aborted at 46, 60 and 63 dpi, respectively. Aborted kids showed no gross abnormalities, although some slight autolysis was observed. This suggests that death occurred shortly before or during abortion. Four goats delivered liveborn kids; two goats delivered one weak kid each at 66 dpi, and two goats delivered a single healthy kid each at 68 and 69 dpi (Table 2). Four control goats delivered liveborn kids between 75 and 79 dpi, which equals a gestation of 150 to 154 days. This is a normal gestation period for goats. In Experiment III, pregnancy outcomes showed similar characteristics (Table 3). Again *C. burnetii* was detected in the placentas of all (ten) goats by IHC and by PCR in the vaginal mucus just after parturition. Three goats aborted (one single kid and two twins) at 52, 59 and 70 dpi; one goat delivered one stillborn and one liveborn weak kid at 69 dpi; two goats delivered one stillborn and one liveborn healthy kid each at 67 and 68 dpi; one goat delivered two weak kids at 62 dpi and three goats delivered healthy kids, all singles at 66, 67 and 68 dpi. The six control goats delivered healthy liveborn kids between 73 and 81 dpi, which equals 149 to 157 days of gestation. Together these results confirm the efficiency of the intranasal inoculation route and indicate abortion as a major manifestation of *C. burnetii* infection. However, the finding that sometimes both healthy and dead kids are delivered by the same doe indicates that the infection is not always lethal for the offspring.

**Table 2:** Pregnancy outcome and *C. burnetii*-specific PCR results using DNA isolated from the indicated tissues taken at necropsy of the kids in Experiment II.

Days post inoculation	14		28		42		46	56		60	63	66	66	68	69	69
Days of gestation	89		103		117		121	131		135	138	141	141	143	144	144
Pregnancy outcome	nec		nec		nec		abo	nec		abo	abo	kid	kid	kid	nec	kid
Number of kids	1	1	2	2	1	3*	2	2	1	2	2	1	1	1	1	1
Goat ID	25	26	27	28	31	32	36	29	30	35	40	33	38	37	34	39
Tissue																
**Foetus 1**																
Foetal membrane	−	−	−	−	+++	++	+++	++	+++	+++	++	+++	++	+++	+++	+++
Placentome/cotyledon	−	−	+	++	+++	+++		+	++	+++	+++	+++	+++	+++	+++	+++
Amniotic fluid	−	−	−	−	+	+		−	+						+	
Allantoic fluid	−	−	−	−	+++	+		+	−						++	
Foetal blood	−	−	−	−	++	+	−	−	−	−	−	−	−	−	+++	−
Spleen	−	−	−	−	+	+	+	+	−	+	+	−	−	+	+	++
Liver	−	−	−	−	+	++	+	−	+	+	+	−	+	+	++	++
Kidney	−	−	−	−	+	+	+	+	+	+	−	−	+	+	+	+
Lung	−	−	−	−	+	++	−	+	+	+	−	−	+	+	+	++
Heart	−	−	−	−	++	++	−	−	+	+	−	+	−	+	++	+
**Foetus 2**																
Foetal membrane			−	+++		−		+		+++	+++					
Placentome/cotyledon			+	++		++		+		+++	+++					
Amniotic fluid			−	−		+		−								
Allantoic fluid			−	++		+		+								
Foetal blood			−	−		+	−	−		+	++					
Spleen			−	−		+	+	−		++	+					
Liver			−	−		++	+	−		++	+					
Kidney			−	−		+	−	−		+	++					
Lung			−	−		+	+	−		++	+					
Heart			−	−		++	+	−		+	++					

nec: necropsy before kidding, abo: abortion, kid: kidding (liveborn kids), “−”: negative result, “+”: 30≤PCR cycle threshold (Ct)<40, “++”: 20≤Ct<30, “+++”: Ct<20, blank: no sample available, * = results of 2 kids shown, third one like other 2.

Results are measurements at the day of necropsy indicated by days post inoculation and days of gestation. Liveborn kids were euthanised on the day of parturition for necropsy. No significant difference between aborted and liveborn kids was observed.

**Table 3:** Results of *C. burnetii*-specific PCR on DNA isolated from the indicated tissues taken at necropsy of the kids in Experiment III.

Days post inoculation	52	59	68	67	94	94	94	98	98
Days post-partum	0	0	0	4	26	26	28	31	31
Status kid	abortion	abortion	abortion	weak	healthy	healthy	healthy	healthy	healthy
Tissue									
**Foetus 1**									
Spleen	+	++	++	++	+	−	−	−	−
Liver	+	++	+	+	−	+	−	−	−
Kidney	−	++	+	+	−	−	−	−	−
Lung	+	++	+	++	−	−	−	−	−
Heart	+	++	−	+	−	−	−	−	−
**Foetus 2**									
Spleen		+							
Liver		++							
Kidney		++							
Lung		++							
Heart		++							

“−”: negative result, “+”: 30≤PCR cycle threshold (Ct)<40, “++”: 20≤Ct<30, blank: no sample available.

*C. burnetii* was detected in the placentas of all Coxiella-inoculated goats by immunohistochemistry and by PCR in the vaginal mucus just after parturition. Healthy kids stayed alive until the end of the experiment. Results are measurements at the day of necropsy indicated by days post inoculation and days post-partum.

### Dissemination of *Coxiella burnetii* in Intranasally Inoculated Goats as Detected with PCR

To study the spread of *C. burnetii* in the goats after intranasal inoculation, two Coxiella-inoculated goats and one control goat were killed each 14^th^ day until 69 dpi in Experiment II. Thereafter, one Coxiella-inoculated goat was killed at 77, 98, 119, 126, 140 and 141 dpi and one control goat was killed at 84, 111 and 140 dpi. Tissues of the respiratory tract, genital tract, haematopoietic system, liver, urinary tract, alimentary tract and from the heart, glandula parotis and perirenal fat were sampled for PCR analysis.

At 14 dpi *C. burnetii* DNA was only detected in the upper respiratory tract of the two necropsied infected goats and in the spleen and thymus of one of these goats. At 28 dpi *C. burnetii* DNA was detected only in the uterus and placenta of the two necropsied goats. At 42 dpi higher amounts of *C. burnetii* DNA in the uterus and placenta were detected reaching Ct values <20. *C. burnetii* DNA was also detected in most other tissues, including the upper and lower respiratory tract, haematopoietic system, liver, urinary and alimentary tract and the heart. In the goats necropsied at 56 dpi comparable *C. burnetii* DNA distributions were detected as in the goats necropsied at 42 dpi, although fewer tissues were positive. At 69 dpi, one goat was necropsied just after kidding, while the other one was in parturition. The quantity of *C. burnetii* DNA in the placenta and uterus of both animals was high (Ct values 14–18). *C. burnetii* DNA was also present in the respiratory, alimentary and urinary tract, the haematopoietic system, liver and in the heart. In the days after parturition the amount of DNA in the goats necropsied decreased and at 98 dpi (32 dpp) most organs tested negative for *C. burnetii* DNA except for the tonsils and mucosa of the upper respiratory tract, the uterus and caruncles, the bladder and gut. *C. burnetii* DNA was detected in the female genital tract until 57 dpp, in the gut until 60 dpp and in the mucosa of the nostrils in the upper respiratory tract until the end of the experiment at 95 dpp. The abdominally taken vaginal mucus swabs were *C. burnetii* DNA positive at 119 and 126 dpi, but negative at 140 and 141 dpi. An overview of the amount of *C. burnetii* DNA in the sampled tissues of the Coxiella-inoculated goats during the experiment is presented in Table 4. In the control goats no *C. burnetii* DNA was detected in any of the tissues at any point in time post inoculation (data not shown). Together, our results indicate that after intranasal inoculation *C. burnetii* DNA is detectable in the upper respiratory tract up to 14 dpi and that *C. burnetii* reached the placenta between 14 and 28 dpi. Then *C. burnetii* appears to spread to different organs with increased DNA levels detectable towards parturition. After parturition, the *C. burnetii* DNA gradually decreases to undetectable levels.

**Table 4:** Results of *C. burnetii*-specific PCR using DNA isolated from the indicated tissues taken at necropsy of Coxiella-inoculated goats.

Days post inoculation	14		28		42		56		69	69	77	98	119	126	140	141
Days post-partum										0	9	32	57	60	81	95
Goat ID	25	26	27	28	31	32	29	30	34	39	37	33	40	38	35	36
Tissue																
**Upper respiratory tract**																
Lymph nodes	+	+	−	−	−	+	−	−	−	+	+	−	+	−	−	−
Mucosa	−	+	−	−	+	+	+	+	+	++	+	+	+	+	+	+
Tonsils	+	+	−	−	−	+	−	+	+	++	+	+	−	−	−	−
**Lower respiratory tract**																
Lymph nodes	−	−	−	−	−	+	−	−	−	+	+	−	−	+	−	−
Bronchi	−	−	−	−	−	+	−	+	+	++	+	−	−	−	−	−
Lung	−	−	−	−	−	−	+	−	−	++	−	−	−	−	−	−
**Female genital tract**																
Udder	−	−	−	−	+	−	−	−	+	+	−	−	−	−	−	−
Lymph nodes udder	−	−	−	−	+	−	−	−	+	+	+	−	−	−	−	−
Ovarium	−	−	−	−	−	+	−	−	+	+	+	−	−	−	−	−
Lymph node iliaca	−	−	−	−	−	+	−	−	+	+	+	−	−	−	−	−
Uterus, non placenta	−	−	−	+	++	++	+	+	++	+++	++	+	+	−	−	−
Placentome/caruncle 1	−	−	+	++	+++	++	+	++	+++	+++	++	+	+	−	−	−
Placentome/caruncle 2			+	++		+++	+						−			
Uterus swab													−	−	−	−
Vaginal mucus, abd. taken													+	+	−	−
**Haematopoietic system**																
Spleen	−	+	−	−	−	+	−	−	−	++	−	−	−	−	−	−
Thymus	−	+	−	−	−	+	−	−	−	++	−	−	−	−	−	−
Bone marrow	−	−	−	−	+	++	−	+	+	+	+	−	−	−	−	−
Blood	−	−	−	−	−	−	−	−	−	−	−	−	−	−	−	−
**Liver**																
Lymph nodes	−	−	−	−	−	+	−	−	−	+	−	−	−	−	−	−
Parenchyma	−	−	−	−	−	−	−	+	−	+	−	−	−	−	−	−
Bile	−	−	−	−	−	+	−	+	−	−	−	−	−	−	−	−
**Urinary tract**																
Kidney	−	−	−	−	−	+	−	+	−	+	+	−	−	−	−	−
Bladder	−	−	−	−	−	−	−	+	+	+	+	+	−	−	−	−
Urine	−	−	−	−		−		+	−	−	−			−	−	−
**Alimentary tract**																
Gut	−	−	−	−	+	+	−	−	+	++	+	+	−	+	−	−
Lymph nodes	−	−	−	−	+	−	−	−	−	+	−	−	−	−	−	−
**Miscellaneous**																
Heart	−	−	−	−	−	+	−	−	−	+	+	−	−	−	−	−
Glandula parotis	−	−	−	−	−	+	−	−	+	++	+	−	−	−	−	−
Perirenal fat	−	−	−	−	+	+	−	−	−	+	+	−	−	+	−	−
**Excretion products**																
Faeces	−	−	−	−	−	−	−	−	++	+	−	+		+	+	+
Vaginal mucus, ext. taken	−	−	−	−	−	−	−	−	+	+++	++	+		+	+	+
Milk											+	−	−	−	−	−

abd. taken: abdominally taken, ext. taken: externally taken, “−”: negative result, “+”: 30≤PCR cycle threshold (Ct)<40, “++”: 20≤Ct<30, “+++”: Ct<20, blank: no sample available.

Results are measurements at the day of necropsy indicated by days post inoculation and days post-partum. Tissues organised per organ system.supp.

### Dissemination of *Coxiella burnetii* in the Goats as Detected by Histopathology

**Figure 1:**
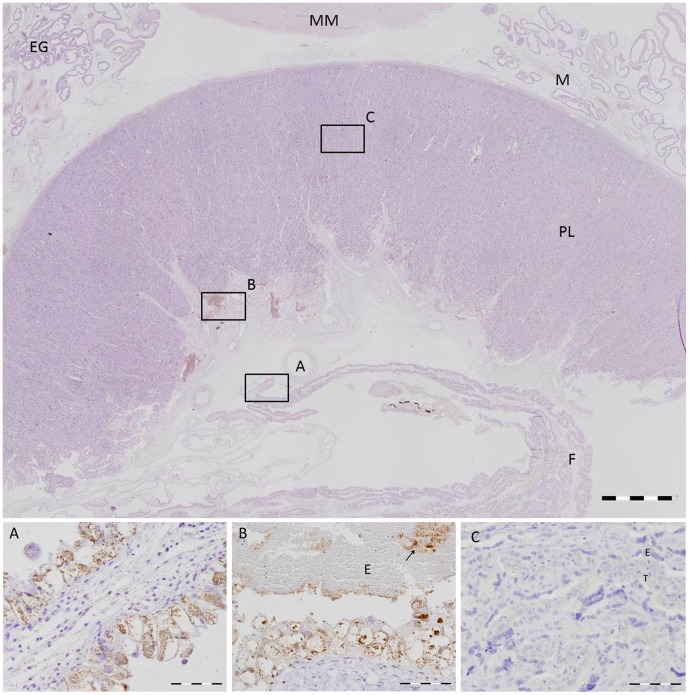
Overview of the placentome of a Coxiella-inoculated goat. Top: Scanned haematoxylin and eosin stained section of the placentome of a goat necropsied at 56 dpi (day 131 of pregnancy). No inflammatory reaction in the maternal endometrium (M), the placentome (PL) or the foetal allantochorion (F). MM = myometrium, EG = endometrial glands. Bar = 1 mm. Bottom: Higher magnification of areas A, B and C depicted by the rectangles in the overview. Serial section immunostained for the presence of *Coxiella burnetii* antigen (brownish colour). A. Foetal allantochorion showing severe swelling of the trophoblast cells caused by the formation of large intracytoplasmic vacuoles. The vacuoles are filled with numerous *C. burnetii* bacteria. Bar = 200 µm. B. Erythrophagous zone showing similar vacuolation and swelling of the trophoblasts at the base of the foetal villi. Numerous *C. burnetii* bacteria are seen within the vacuoles of the trophoblasts and in the blood-filled lacuna between the foetal and maternal epithelium (arrow). E = erythrocytes. Bar = 200 µm. C. Placentome. Absence of *C. burnetii* bacteria in the synepitheliochorial placenta. The maternal epithelium (E) and foetal trophoblasts (T) show no morphological alterations. Bar = 100 µm.

**Figure 2:**
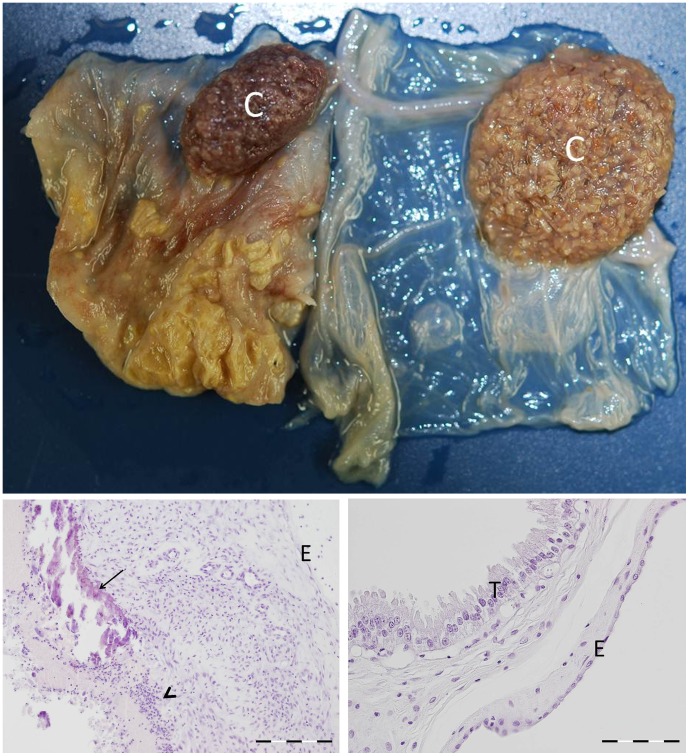
Comparison of a *C. burnetii* positive placenta and a *C. burnetii* negative placenta at parturition. Top. Formalin-fixed allantochorion of an aborted kid (left) and the allantochorion of a kid from a control goat (right). The intercotyledonary allantochorion of the aborted kid is severely thickened and dull with a yellow-brownish exudate, while the cotyledon (C) shows no obvious macroscopic changes. The allantochorion of the control kid on the right is thin, glistening and transparent. Bottom. Left. Haematoxylin- and eosin-stained section of the intercotyledonary allantochorion of an aborted kid. Notice the severe inflammatory changes in the stroma of the allantochorion. The trophoblast layer is lost with dystrophic calcification of necrotic tissue (arrow) and a purulent exudate (arrowhead). Bar = 200 µm. Right. Histological appearance of a normal allantochorion of a kid from a control goat. The trophoblast layer is intact with a normal appearance of the trophoblast cells (T). Low cellularity in the stroma. E = endothelium of the allantochorion. Bar = 100 µm.

**Figure 3:**
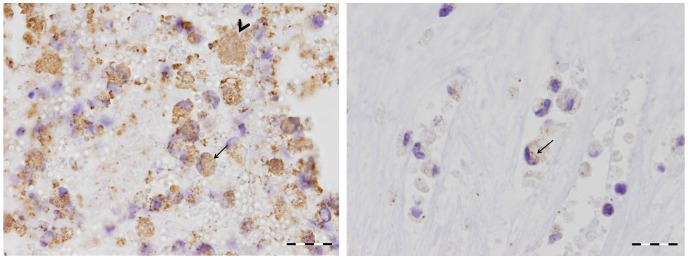
Close up of the allantochorion of an aborted kid due to *C. burnetii* infection. The allantochorion of an aborted kid is immunostained to detect the presence of *C. burnetii* antigen. On the left: Numerous macrophages are present in the necropurulent exudate that covers the allantochorion. Both macrophages (arrow) and sloughed trophoblasts (arrow head) are filled with *C. burnetii*. On the right: macrophages in the stroma of the allantochorion have phagocytised *C. burnetii* bacteria (arrow). Bar = 20 µm.

Tissues sampled for PCR were also investigated for histopathology. At 14 dpi neither histopathological lesions nor *C. burnetii* antigen were detected in any of the tissues of the Coxiella-inoculated goats. The first histopathological changes were seen in the foetal part of the placenta of one of the two goats killed at 28 dpi. The trophoblasts of the allantochorion appeared swollen and contained a large vacuole that dislocated the nucleus to the periphery of the cells, leading to a crescent cell shape. Immunohistochemistry (IHC) demonstrated numerous *C. burnetii* bacteria within the vacuoles of the trophoblasts. At this stage only the trophoblasts of the intercotyledonary allantochorion were affected, but at 42 and 56 dpi the erythrophagic trophoblasts at the base of the cotyledonary villi were gradually affected as well, starting from the periphery of the placentome. At 56 dpi all trophoblasts were loaded with large quantities of bacteria. However, *C. burnetii* antigen was never detected in the trophoblasts covering the cotyledonary villi, which form the true placenta together with the uterine epithelial cells of the maternal crypts (Figure 1). This indicates that the nutrient and gas exchange with the foetus was not disturbed. No inflammatory changes were seen in the allantochorion up to 56 dpi. At 69 dpi, one goat was necropsied just after kidding while the other one that was necropsied was in parturition. The allantochorion of both of these animals was severely thickened and leathery with a yellow/brownish exudate. The placentas of all *C. burnetii*-inoculated goats that aborted or delivered normally showed this appearance. These inflammatory changes were mainly confined to the intercotyledonary region of the placenta, while most cotyledons appeared normal macroscopically. Histologically, a heavy purulent to necropurulent inflammation was seen with large necrotic areas within the trophoblast layer, often accompanied by dystrophic calcification (Figure 2). The inflammatory response in the allantochorionic stroma consisted mainly of polymorphonuclear granulocytes and macrophages, with only a few lymphocytes or plasma cells. Occasionally, thrombosis was seen in blood vessels of the allantochorion. Although most of the trophoblast layer was ablated, numerous Coxiella bacteria were detected in the sloughed debris, in macrophages (Figure 3) and in areas where the trophoblast layer was still intact. In particular, the trophoblast layer of the former haemophagous zone at the base of the cotyledonary villi was often intact and contained large quantities of *C. burnetii*. This may therefore be the most suitable location to detect *C. burnetii* in a placenta after abortion or parturition. No *C. burnetii* antigen was detected in the endometrium or the maternal placental stroma or epithelium at any time during the infection. Compared to the control goats, there was also no significant increase in inflammatory cells in the endometrium until the time of parturition. Only at 9 dpp, was more infiltration of mononuclear cells (macrophages, lymphocytes and plasma cells) and polymorphonuclear granulocytes seen in the endometrium and caruncles as compared to the control goat euthanised at 9 dpp. However, this difference disappeared at later stages post-partum and both Coxiella-inoculated goats and control goats showed a similar degree of inflammatory infiltration associated with the ablation of the maternal placenta and involution of the uterus. Apart from the placenta, no histopathological lesions or *C. burnetii* antigen were detected in any of the other organs examined. Thus even in the tissues in which *C. burnetii* DNA could be detected by PCR, no *C. burnetii* antigen could be detected with immunohistochemistry. As expected, *C. burnetii* antigen was never detected in any of the tissues or placentas of the control animals. Overall, our data indicate that the trophoblasts of the allantochorion are the primary target cells of *C. burnetii* and that replication starts between 14 and 28 dpi. *C. burnetii* does not appear to infect the trophoblasts covering the cotyledonary villi and we found no evidence of *C. burnetii* replication in other tissues than the placenta.

### Dissemination of *Coxiella burnetii* in the Foetuses and Kids

The presence of *C. burnetii* in mainly placental tissues raises the question as to whether the pathogen damages foetal tissues. To address this point, the dissemination of *C. burnetii* in aborted and euthanised liveborn kids was studied. Spleen, liver, kidney, lung and heart tissue were examined for histopathology and isolated DNA from these tissues was subjected to PCR. The investigations in Experiment II focused on the dissemination during pregnancy and at the time of parturition. In Experiment III the liveborn kids from infected does were kept alive to gain insight in the dissemination during the first weeks of their lives.

At 14 dpi, *C. burnetii* DNA was not detected in either the foetal part of the placenta or the foetal organs. At 28 dpi *C. burnetii* DNA was detected in the foetal allantochorion, the placentomes and the allantoic fluid. From 42 dpi until the end of pregnancy high quantities of *C. burnetii* DNA were detected in the foetal parts of the placenta of at least one of the kids. For most foetuses, amniotic and allantoic fluids as well as samples from the foetal spleen, liver, kidney, lung and heart tested positive for *C. burnetii* DNA. No significant difference in results was found between the placentas and foetal organs of aborted and liveborn kids. Therefore the placenta from liveborn kids also contained high amounts of *C. burnetii*. Histopathological examination of the tissues demonstrated a slight granulomatous hepatitis in some of the kids, but this was also present in kids from the control group. *C. burnetii* antigen was not detected by IHC in any of the sampled tissues. Two liveborn kids in Experiment III necropsied at 26 dpp were positive for *C. burnetii* DNA in either the spleen or liver. The three kids necropsied at 28 and 31 dpp were negative for *C. burnetii* DNA in all sampled organs. Tissues of all foetuses and kids born from the control goats tested negative for *C. burnetii* DNA. Detailed information about the amount of *C. burnetii* DNA in the sampled tissues detected by PCR of the foetuses of *C. burnetii*-inoculated goats is shown in Tables 2 and 3. It is important to note that the *C. burnetii* load in the placentas of aborted and liveborn kids were similar. After parturition, organs of kids initially contain high amounts of *C. burnetii*, but tested negative after 28 days.

### Excretion of *Coxiella burnetii* in Faeces, Vaginal Mucous, Blood, and Milk

To investigate the excretion of *C. burnetii* in faeces, vaginal mucus, blood and milk, Coxiella-inoculated and control goats were frequently sampled right from the start of the experiment. Milk sampling started just after parturition. Blood samples were taken to detect bacteraemia in particular after inoculation and parturition. Samples were examined by PCR for the presence of *C. burnetii* DNA.

**Figure 4:**
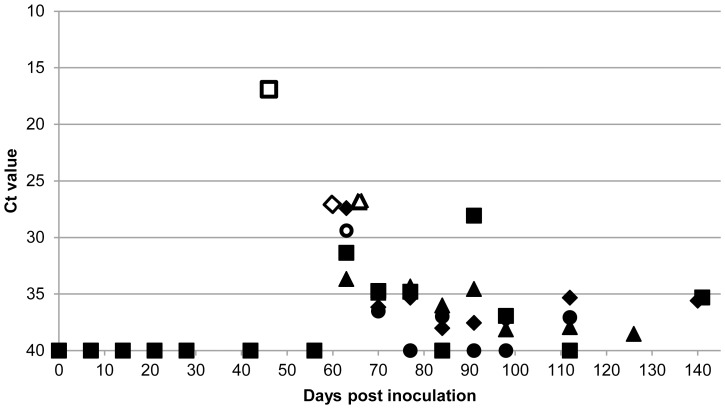
Detection of *C. burnetii* DNA in the faeces of Coxiella-inoculated goats over time. Detection of *C. burnetii* DNA in the faeces of four challenged goats of which a complete sampling sequence was present from inoculation until 119, 126, 140 and 141 days post inoculation (dpi). Goat ID 35: ⧫, 36: ▪, 38: ▴, 40: •. Faecal samples were taken at the indicated dpi and *C. burnetii* DNA was measured by PCR. Ct value of 40 is negative, Ct value <40 is positive. Parturition days are indicated as open symbols. Up until 42 dpi and on 56 dpi the four goats were negative. At 56 dpi three other goats of the group were positive before their parturition (data not shown). Data indicate that *C. burnetii* was detected in the faeces after parturition.

**Figure 5:**
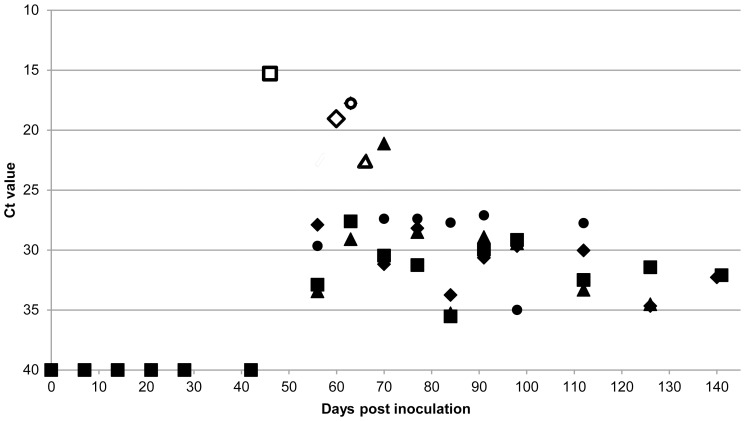
Detection of *C. burnetii* DNA in vaginal mucus of Coxiella-inoculated goats over time. Detection of *C. burnetii* DNA in vaginal mucus of four challenged goats of which a complete sampling sequence was present from inoculation until 119, 126, 140 and 141 days post inoculation (dpi). Goat ID 35: ⧫, 36: ▪, 38: ▴, 40: •. Vaginal mucus samples were taken at the indicated dpi and *C. burnetii* DNA was measured by PCR. Ct value of 40 is negative, Ct value <40 is positive. Parturition days are indicated as open symbols. Until 42 dpi the four goats were negative. Data indicate that *C. burnetii* was detected in the vaginal mucus after the first parturition in the group.

All excreta and blood samples from the control goats remained negative for *C. burnetii* DNA for the entire duration of the experiment. For the Coxiella-inoculated goats, *C. burnetii* DNA was never detected in any of the faecal or vaginal mucus samples until the first abortion (goat ID 36) at 46 dpi. At the first sampling point after the first abortion (56 dpi) three of the eight goats tested positive for *C. burnetii* DNA in their faeces, while at 63 dpi (after the second and third abortions) the faeces of all eight goats tested positive. For the vaginal mucus samples this trend was even more prominent. *C. burnetii* DNA was first detected in vaginal mucus in the first goat that aborted, while at the next sampling point (56 dpi), all eight goats already contained *C. burnetii* DNA in their vaginal mucus. This strongly suggests that *C. burnetii* is not excreted in the faeces or vaginal mucus before parturition. Monitoring of samples after parturition showed that *C. burnetii* DNA remained present in the faeces and the vaginal mucus of the goats until the end of the experiment. The results of faeces and vaginal mucus sampling of four goats that were sampled until 119 and 126 dpi and until the end of the experiment at 141 dpi, are presented in Figure 4 and Figure 5, respectively.

**Figure 6:**
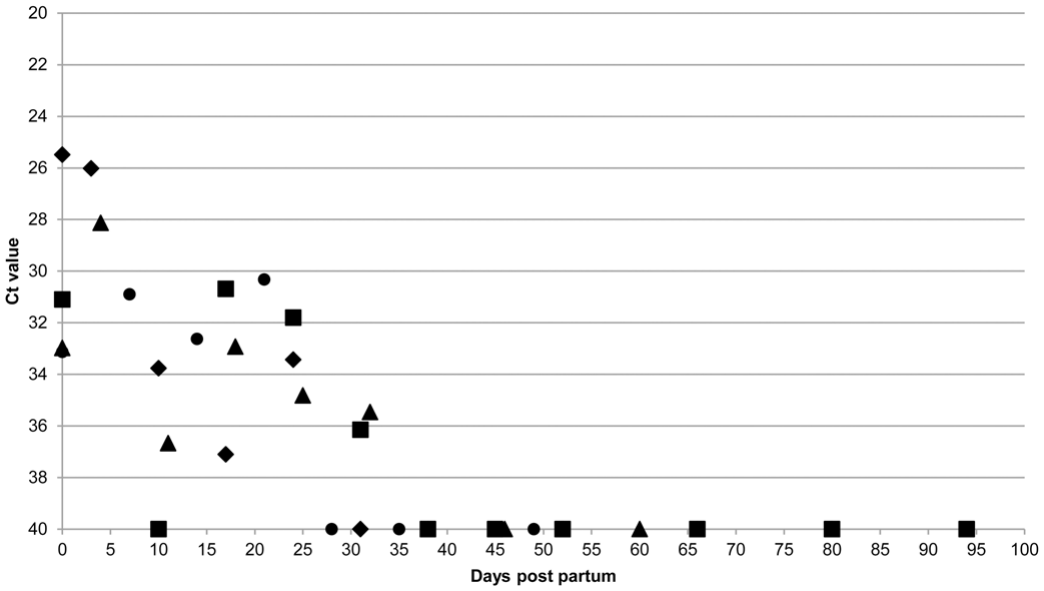
Detection of *C. burnetii* DNA in milk of Coxiella-inoculated goats over time. Detection of *C. burnetii* DNA in milk of four challenged goats of which a complete sampling sequence was present from inoculation until 119, 126, 140 and 141 days post inoculation (dpi). Goat ID 35: ⧫, 36: ▪, 38: ▴, 40: •. Milk samples were taken at the indicated days post-partum and *C. burnetii* DNA was measured by PCR. Ct value of 40 is negative, Ct value <40 is positive. After parturition *C. burnetii* DNA was detected in the milk until 32 days post-partum.

Analysis of the blood and milk samples only revealed *C. burnetii* DNA in the blood after the first abortion. Three goats had only one positive sample at 49 dpi, one goat had two positive samples (at 49 and 67 dpi) and goat 36 had three positive blood samples at 46, 48 and 49 dpi. *C. burnetii* DNA was detected in the milk of the Coxiella-inoculated goats after parturition until 38 dpp. Milk samples from later dates remained negative until the end of the experiment. The results of milk sampling of four goats that were followed until 119 and 126 dpi or until the end of the experiment at 141 dpi, are detailed in Figure 6.

### Detection of *Coxiella burnetii* in the Environment

**Figure 7:**
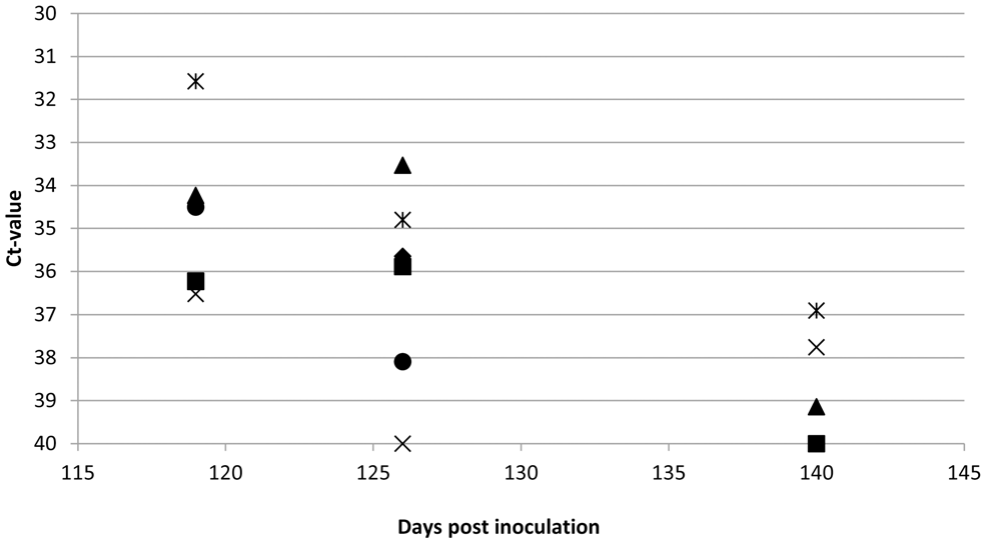
*C. burnetii*-specific PCR results of samples from the environment of Coxiella-inoculated goats. *C. burnetii* PCR results of samples from the environment of the challenged goats measured at three time points after inoculation. ⧫: clean wood shavings stored in the facility, ▪: used wood shavings, ▴: swab from the slip mat, ×: swab from the air, ?: swab from the water, •: air filter. Ct value of 40 is negative, Ct value <40 is positive. Results indicate an environmental contamination of *C. burnetii* DNA at the end of the experiment.

To investigate the possible *C. burnetii* contamination of the box environment, samples were taken from bedding material, floor, water and the air. The results of the environmental samples obtained in Experiment II are shown in Figure 7. Samples taken from 119 dpi onwards were positive for *C. burnetii* DNA and remained positive (albeit at lower levels) until the end of the experiment. The air samples from one of the two groups of *C. burnetii*-inoculated goats (Group C) in Experiment III were negative until 57 dpi. At 70 dpi the filter was positive for *C. burnetii* DNA (Ct value 27). This signal was lower at 77 dpi (Ct value 31) and at 84 dpi (Ct value 33). The first parturition in this group occurred at 62 dpi. These results indicate that *C. burnetii* may first spread into the environment after parturition and that the DNA persists until the end of the experiment.

## Discussion

The goal of this study was to investigate the dissemination and excretion of *C. burnetii* in pregnant goats before and after parturition. To closely mimic the field situation, a natural inoculation route was preferred. In goat herds, inhalation of *C. burnetii* contaminated aerosols and oral uptake from the environment are the most likely routes of infection. As these routes of inoculation had not previously been investigated in experimental settings, we first tested the ability to infect goats via the oral and intranasal route and compared this with results from the frequently used subcutaneous route of inoculation [18], [19], [21]. Our results indicate that the intranasal inoculation route is most effective in infecting pregnant goats with *C. burnetii*. The high efficiency of intranasal inoculation was reproducible in two successive experiments and resembles results obtained after subcutaneous inoculation [18], [19], [21]. As the route of inoculation (intranasal versus subcutaneous) may influence the pathogenesis of Q fever and excretion of *C. burnetii,* we used the more natural intranasal inoculation route in our experiments.

Our results indicate that after intranasal inoculation, trophoblasts in the allantochorion of the placenta are the primary target cells for *C. burnetii*. *C. burnetii* was first detected by PCR and IHC at 28 dpi but not at 14 dpi. This suggests that it may take between 2 to 4 weeks for *C. burnetii* to multiply to detectable levels in the trophoblasts after entering the body. This time line corresponds with the results in which subcutaneous inoculation was used for challenge and in which *C. burnetii* was detected in the trophoblasts at 26 dpi [19]. A field observation suggests the progression from infection to abortion could be as short as 21 days [22]. From 42 dpi up to the time of parturition, the number of tissues that tested positive for *C. burnetii* DNA and the amount of DNA detected increased. The increase in the amount of *C. burnetii* DNA, however, does not seem to be caused by replication of *C. burnetii* in the parenchyma of the tissues. We were unable to detect *C. burnetii* antigen by IHC outside the placenta and had no histological indications for *C. burnetii* replication or inflammation. After parturition the amount of *C. burnetii* DNA in the tissues gradually decreased to zero, except for the mucosa of the nostrils. This decrease nicely reflects the trophoblast tropism of *C. burnetii*. With parturition these cells are removed from the body, depriving *C. burnetii* of its replication niche. The observed sequence of dissemination of *C. burnetii* in goats seems to resemble those after subcutaneous inoculation [18], [19]. This indicates that the inoculation route is not a major factor in the dissemination and replication of *C. burnetii* in pregnant goats. The tropism of *C. burnetii* for trophoblasts suggests that only pregnant animals are susceptible to *C. burnetii* infection. It is not clear if they should be pregnant to become infected. We cannot exclude the possibility that following infection, undetectable numbers of *C. burnetii* can hide in the body to infect trophoblasts when they become available [23].

An important finding of our study is that *C. burnetii* infection of pregnant goats does not always result in abortion. After intranasal inoculation, several infected goats gave birth to healthy liveborn kids although abortion was observed as well. Also the birth of stillborn and liveborn kids from the same doe indicates that *C. burnetii* infection is not always lethal for kids. This is the first time that the birth of liveborn kids has been confirmed under experimental conditions. Until now, only abortions and pre-term weak kids were reported in goats under experimental conditions after subcutaneous challenge with *C. burnetii* [18], [19], [21]. Our study outcome reflects the natural situation in *C. burnetii*-infected herds where abortions (up to 80%), weak-born kids as well as liveborn kids have been registered [9], [14], [16], [20], [24]. Remarkably, our results show no relation between pregnancy outcome and excretion of *C. burnetii* with the placentas in infected pregnant goats. Also the birth of newborn kids from infected goats was accompanied by high amounts of *C. burnetii* being excreted with the placenta. This is fully consistent with the field observation that demonstrated *C. burnetii* can be present in a placenta from a goat that delivers normally [16]. However, it challenges the suggestion that fewer bacteria are excreted when healthy kids are borne [25]. The consequence of our finding is that, besides abortions, normal deliveries in infected goats contribute to the environmental contamination and should therefore be considered as a major zoonotic risk for humans.

The birth of liveborn kids from animals with a heavily infected placenta may be explained by the histopathological changes in the placenta after inoculation. *C. burnetii* was not detected in the trophoblasts covering the cotyledonary villi involved in the exchange of gases and nutrients, which may prevent premature foetal death. Instead, foetuses may either die shortly before or during abortion, or may be born alive. This situation is different in caprine Brucella and Chlamydia infections. In these two types of infections, alterations in the foetoplacental binding lead to foetal death culminating in abortion [26], [27], [28], [29]. The factors that determine the foetal fate in *C. burnetii* infected goats have yet to be determined.

Live kids delivered from infected goats contained *C. burnetii* DNA in several organs. However, no *C. burnetii* antigen could be detected. Therefore, like others [19], we have no indication for replication of *C. burnetii* in these organs. During the first weeks of life, the level of *C. burnetii* DNA positivity in the tested organs decreased to undetectable levels within 28 days. This clearance was also detected in the does, but finished earlier. These data suggest that *C. burnetii* is effectively cleared from the kid’s tissues during the first weeks post-partum. However, the possibility of a persistent infection or carrier state with *C. burnetii* hiding in organs under the detection limit of the PCR cannot be excluded.

We found no evidence that *C. burnetii* is excreted in the faeces and vaginal mucus before parturition. Moreover, we found no indications for active replication of *C. burnetii* in the gut, liver or genital tract (besides the trophoblasts) which could lead to active excretion of *C. burnetii* in faeces and vaginal mucus before and after parturition. These results deviate from the general assumption that *C. burnetii* is excreted in faeces and vaginal mucus before parturition [6], [13]. In our study faecal and vaginal mucus samples only tested positive for *C. burnetii* DNA after the first abortion in the group. After this parturition, *C. burnetii* DNA remained present in the faeces and vaginal mucus until the end of the experiment. It is possible that parturition, with the excretion of high numbers of *C. burnetii,* influences the test result either directly or via contamination of the environment. The absence of *C. burnetii* DNA in abdominally taken vaginal mucus swabs compared to the presence of *C. burnetii* DNA in vaginal mucus swabs taken externally on the same day may point to an external source of *C. burnetii*. The presence of *C. burnetii* in the environment of the infected goats after parturition may be a source of contamination as suggested by Welsh *et al* [30]. All things being considered, we favour the hypothesis that *C. burnetii* is not excreted before parturition in *Coxiella-inoculated* pregnant goats and that excretion of *C. burnetii* in faeces and vaginal mucus after parturition may be indirect following contamination by the environment.

Post-partum, all Coxiella-inoculated goats excreted *C. burnetii* DNA in the milk. Excretion stopped after 38 dpp. Arricau-Bouvery *et al* [18] detected *C. burnetii* DNA in the milk until at least 52 days after abortion. This difference with the present study can be explained by differences in milking pattern; Arricau-Bouvery *et al* [18] did not milk the goats, but only sampled them, so *C. burnetii* could accumulate in the milk in the mammary gland. In the present study the goats were milked daily so *C. burnetii* was discharged from the udder. Although the goats were milked in the *C. burnetii-* contaminated box environment, we were able to obtain negative results. These aseptically taken individual milk samples do not appear to have been influenced by the presence of environmental *C. burnetii*.

In summary, this study showed the intranasal inoculation route of *C. burnetii* to be effective in infecting pregnant goats. Inoculation results in strong tropism of *C. burnetii* towards the placenta and replication occurred in the trophoblasts of the placenta, but not in other tissues of goats and kids. Importantly, *C. burnetii* infection does not always result in abortion as infected goats gave birth to live and dead kids. The main excretion route of *C. burnetii* from infected pregnant goats is during abortion and during the delivery of kids. This suggests that normal parturitions in an infected herd may also form a source of infection.

## Materials and Methods

### Ethics Statement

All animal experiments were approved by the Animal Experiment Commission of the Central Veterinary Institute, part of Wageningen UR, in accordance with the Dutch regulations on animal experimentation (registration numbers 2009082.c, 2009079.a, 2010098.d and 2011111.c). Everything possible was done to minimise animal suffering. Humane endpoints were defined in advance. Whenever these endpoints were reached, animals were euthanised.

### Inoculum

*C. burnetii* strain X09003262-001 was isolated from a placenta of one of the 25% of the goats that aborted on a farm during the Q fever outbreak in the Netherlands (farm N, [11]). The presence of *C. burnetii* in the placenta was confirmed by immunohistochemical (IHC) staining [20] and *C. burnetii*-specific quantitative polymerase chain reaction (qPCR) [11]. The strain was genotyped as CbNL01, the predominant *C. burnetii* genotype in the Dutch Q fever outbreak [9], [12]. *C. burnetii* was isolated by crushing a part of the placenta from the allantochorion and base of the cotyledon in a ribolyser (FastPrep-24, MP Biomedicals (USA), lysing matrix D with ¼ ceramic beads, 2 times 20 sec with 6 m/sec^2^ and a 5 min break in between). This lysate was filtered stepwise using a cell strainer (Nunc, Denmark) and filters with pore sizes of 1.2 µm and 0.45 µm (Pall Cooperation, USA). Filtered material was inoculated onto a culture of Buffalo Green Monkey (BGM) cells (European Collection of Cell Cultures) with culture medium without antibiotics (EMEM with 10% bovine serum albumin, 1% NEAA, 1% glutamax) and incubated for 14 days at 37°C in a closed flask. Culture medium was refreshed twice a week. Growth of *C. burnetii* was monitored by vacuolisation of the BGM cells and confirmed by an immunofluorescence assay using a *C. burnetii*-specific monoclonal antibody (MAB313-oregon green, Squarix) and PCR. Cell culture was confirmed negative for *Chlamydia abortus* by qPCR targeting the OmpA gene (forward primer 5′-CTCCTTACAAGCCTTGCCTGTAG-3′; reverse primer 5′-CCTGAAGCACCTTCCCACAT-3′; probe: FAM labelled 5′-CCAGCTGAACCAAGTTTATTAATCGATGGCA-3′), *Simkania negevensis* (CVI in-house PCR: forward primer 5′-GTTACGAGCCTGGCGATGCCA-3′; reverse primer 5′-AAAGTTGCTTGGCTGCGCGG-3′) and mycoplasma (forward primer 5'- GGGAGCAAACAGGATTAGATACCCT-3′; reverse primer 5'- TGCACCATCTGTCACTCTGTTAACCTC-3). A large batch of strain X09003262-001 was prepared as follows. The infected monolayer from the primary isolation was frozen at −80°C for 30 min, thawed and then monolayer cell line debris was removed by centrifugation (10 min, 100×*g*). The supernatant was added to BGM cells. After confirmed growth, the supernatant was collected and cell debris was removed as described above. The supernatant was stored in aliquots of 1 ml at −80°C. The mouse infective dose (MID) of the batch was determined as described by Arricau-Bouvery *et al* [18]. In brief, four mice (Swiss random OF1, 2 month of age) were inoculated intraperitoneally with decimal dilutions of *C. burnetii* strain X09003262-001. After 9 days the mice were euthanised and *C. burnetii* was detected in the spleen by PCR. The MID was defined as the maximal decimal dilution that infected all mice in the group. Before inoculation, the inoculum was adjusted to the required MID by dilution with culture medium.

### Animal experiments

#### Experiment I

Twelve healthy, pregnant, serologically *C. burnetii* negative Dutch dairy goats were purchased from a Dutch dairy goat farm without a known history of Q fever. All goats tested negative for antibodies against *C. burnetii* (LSIVET RUMINANT milk/serum Q-fever ELISA kit, LSI, France) and vaginal mucus tested negative for *C. burnetii* DNA and for *Chlamydia abortus* DNA by PCRs on the day of arrival. Two goats served as negative controls and were housed in animal biosafety level (aBSL)2 facilities. Five groups of two goats were housed in aBSL3 facilities. Each group of two goats was inoculated on day 90 of gestation, either orally with 10^4^ or 10^6^ MID, intranasally with 10^4^ or 10^6^ MID, or subcutaneously with 10^4^ MID. Intranasal inoculation was performed during forced inhalation with a nozzle in the left nostril with the right nostril being held closed. Subcutaneous inoculation was performed just in front of the shoulder. Goats were monitored daily for general health via clinical inspection with an emphasis on behaviour, appetite and consistency of the faeces. Goats were considered infected with *C. burnetii* when *C. burnetii*-confirmed abortion occurred or the placenta was tested positive for *C. burnetii* by PCR.

#### Experiment II

Twenty-seven healthy pregnant serologically *C. burnetii* negative Alpine yearling goats were purchased from INRA (Institut National de la Recherche Agronomique, Domaine de Galle), France. Pregnancy and the duration of pregnancy were confirmed by echography. All goats tested serologically negative for antibodies against *C. burnetii* (LSIVET RUMINANT milk/serum Q-fever ELISA kit, LSI, France) and *Chlamydia abortus* (Chekit Chlamydophila abortus antibody test kit, IDEXX Laboratories B.V., the Netherlands) on the day of arrival. Nine negative control goats were housed in aBSL2 facilities. Two groups of eight goats (Groups A and B) were separately housed in two aBSL3 facilities. On day 76 of pregnancy, the 16 goats of Groups A and B were intranasally inoculated with 1 ml of culture medium containing 10^6^ MID of *C. burnetii,* while the nine negative control animals were intranasally inoculated with 1 ml of culture medium. General health was monitored by rectal temperature and daily clinical inspection with an emphasis on behaviour, appetite and consistency of the faeces.

#### Sampling

Dates of sampling of biological specimens are indicated as days post inoculation (dpi). Sampling dates after parturition are indicated as days post parturition (dpp). Every 14 days (until 69 dpi) two Coxiella-inoculated goats and one control goat in the same stage of pregnancy were euthanised for necropsy. Goats were euthanised at one week and one, two and three months after parturition for necropsy i.e. (depending on their exact parturition date) at 77, 98, 119, 126, 140 and 141 dpi (9, 32, 57, 60, 81 and 95 dpp) for the Coxiella-inoculated goats, and at 84, 111 and 140 dpi (9, 32 and 63 dpp) for the control goats. Kids from the Coxiella-inoculated goats were euthanised on the day of parturition for necropsy; kids from the control goats were euthanised together with their does. Goats and kids were euthanised by exsanguination following intravenous injection of sodium pentobarbital. Two tissue samples from the goat’s respiratory tract (lymph nodes, mucosa, tonsils, bronchia and lung), genital tract (lymph nodes, udder, ovarium, non-placental uterus, placentome, caruncle, uterus mucus and vaginal mucus), haematopoietic system (spleen, thymus, bone marrow, blood), liver (lymph nodes, parenchyma and bile), urinary tract (kidney, bladder and urine), alimentary tract (lymph nodes, ileum and colon) and heart, glandula parotis and perirenal fat were collected with sterilised instruments. Samples were likewise connected from the kid’s spleen, liver, kidney, lung and heart. One sample was stored at −20°C for PCR analysis and the other was fixed in 10% phosphate buffered formalin for histopathology. For each tissue sample a new set of sterilised instruments was used to prevent cross-contamination with *C. burnetii*. Tissues with expected high numbers of *C. burnetii* (e.g. placenta) were handled at the end of the necropsy. At 119, 126, 140 and 141 dpi vaginal mucus swabs were taken from the goats via the abdomen. For this an incision was made through the peritoneum and perineum into the vagina.

Rectal faecal samples and vaginal mucus samples, obtained via the vulva, were taken aseptically from all goats starting on day 0 just before inoculation and at weekly intervals until 28 dpi, every 14 days until 56 dpi, weekly until the end of the experiment and on the day of the parturition. Jugular EDTA blood was taken on day 0 before inoculation, at 1, 2, 3, 5, 7, 9, 10, 12, 14, 16, 21, 28, 42 dpi, then at weekly intervals until the end of the experiment, and at 0, 1, 2, 4, 7, 14 and 21 dpp. After parturition, challenged goats were machine milked once a day. The control goats were not machine milked as they nursed their kids. Milk samples were taken weekly and aseptically from the day of parturition until the end of the experiment for PCR analysis.

Environmental samples were taken by swabbing clean and used wood shavings and slip mats, by sampling water and air using swabs and filters (MD 8 airscan Air Sampler, Sartorius, Germany) at 119, 126 and 140 dpi. Samples were analysed with PCR.

#### Experiment III

The origin and health status of the goats and the setup of this experiment were similar to that described for Experiment II except that six negative control goats and ten goats for intranasal inoculation (10^6^ MID of *C. burnetii*) were included (Groups C and D). Goats were euthanised at 91 dpi (negative controls), at 94 dpi (Group C) or at 98 dpi (Group D). After parturition placental tissue was preserved for histopathology and vaginal mucus was collected for PCR analysis. Weak-born kids were euthanised for ethical reasons, when appropriate. Liveborn kids were kept together with their does until the end of the experiment, when they were euthanised. Kids were necropsied after death. Spleen, liver, kidney, lung and heart were collected as described in Experiment II. The air in the box of Group C was sampled by 8 min filtering at weekly intervals from 14 dpi until 84 dpi (except at 63 dpi). Samples were analysed with PCR.

### Histopathology

For histology and immunohistochemistry (IHC) tissues were fixed, dehydrated, cleared, and processed into paraffin blocks. Sections of 4 µm were cut and collected on silane-coated glass slides. Sections were routinely stained with haematoxylin/eosin and immunostained for the presence of *C. burnetii* antigen. For immunostaining, sections were first autoclaved in citrate buffer (pH 6) for 15 min and then incubated (60 min) with a *C. burnetii*-specific monoclonal antibody (MAB313, Squarix, 1/100 dilution in phosphate buffered saline containing 1% bovine serum albumin and 1% normal goat cotyledon). After washing, sections were incubated with HRP-conjugated Envision™ anti-Mouse-Ig and colour was developed during 5 min with DAB^+^chromogen (Dakopatts, Denmark). Sections were counterstained with haematoxylin for 30 sec, dehydrated and mounted in Eukitt (Kindler, Germany).

### PCR for Detection of *Coxiella burnetii*

DNA was extracted from tissues (20 mg), faeces (20 mg), vaginal mucus swabs (swab tip), EDTA blood (200 µL), milk (200 µL) and environmental samples (swab tip, filter part the size of a swab tip) using a DNA tissue kit (DNeasy Blood&Tissue Kit, Qiagen, the Netherlands) according to the manufacturer’s instructions. All samples were subjected to a quantitative PCR (qPCR) targeting a single copy gene encoding a *C. burnetii*-specific hypothetical protein (gene bank number AY502846) using the forward primer 5'-ATAGCGCCAATCGAAATGGT-3', the reverse primer 5'-CTTGAATACCCATCCCGAAGTC-3', and the NED-labelled probe 5'-CCCAGTAGGGCAGAAGACGTTCCCC-3'. An inhibition control (IC) was constructed using primers for the IS1111a element and a dedicated VIC-labelled probe, as previously published [11]. PCR was performed on a 7500 Fast Real Time PCR system (Applied Biosystems, USA), using 400 nmol/L of primers and 200 nmol/L of probes in 7 µL PerfeCTa Multiplex qPCR Super mix, UNG (2X) with Low Rox dye (Quanta Biosciences, USA), 1 µL of IC, 5 µL of sample and 7 µL of water. An initial UDG incubation for 5 min at 45°C and denaturation/activation for 60 sec at 95°C was followed by 50 cycles of denaturation for 10 sec at 95°C and annealing for 30 sec at 60°C. The detection limit of the PCR was determined at 10 copies for faecal, vaginal mucus, milk and environmental samples and at 100 copies for EDTA blood and tissue samples. For tissues, PCR results were scored negative (-) when the generated PCR cycle threshold (Ct) value was 40 or more; positive (+) for Ct values between 30 and 40, (++) for Ct values between 20 and 30, and (+++) for Ct values below 20. For faecal, vaginal mucus, milk and environmental samples a negative result (Ct value of 40 or more) was scored as Ct 40, a positive result (Ct<40) scored by the generated Ct value.
